# Cocoa Flavanol Supplementation and the Effect on Insulin Resistance in Females Who Are Overweight or Obese: A Randomized, Placebo-Controlled Trial

**DOI:** 10.3390/nu15030565

**Published:** 2023-01-21

**Authors:** Elizabeth J. Simpson, Buddhike Mendis, Mandy Dunlop, Hagen Schroeter, Catherine Kwik-Uribe, Ian A. Macdonald

**Affiliations:** 1 MRC/ARUK Centre for Musculoskeletal Ageing Research, School of Life Sciences, Queen’s Medical Centre, University of Nottingham, Nottingham NG7 2UH, UK; 2 National Institute for Health and Care Research (NIHR), Nottingham Biomedical Research Centre, Queen’s Medical Centre, University of Nottingham, Nottingham NG7 2UH, UK; 3 Department of Nutrition, University of California, One Shields Avenue, 3150E Meyer Hall, Davis, CA 95616, USA; 4 Mars Incorporated, 6885 Elm Street, Mclean, VA 22101, USA

**Keywords:** insulin resistance, cocoa flavanols, females, hyperinsulinemic clamp, HOMA-IR

## Abstract

There is interest in the impact that dietary interventions can have on preventing the transition from insulin resistance to type 2 diabetes, including a suggestion that the bioactive components of cocoa may enhance fasting insulin sensitivity. However, a role for cocoa flavanols (CF) in reducing insulin resistance in the insulin-stimulated state, an important risk factor for cardiovascular disease, is unresolved. This study investigated whether CF consumption improved whole-body insulin-mediated glucose uptake (‘M’) in females with overweight/obesity, using a randomized, double-blinded, placebo-controlled, parallel-group design. Thirty-two premenopausal females (19–49 years; 27–35 kg·m^−2^) with elevated HOMA-IR (HOMA-IR >1.5) supplemented their habitual diet with two servings/day of a high-flavanol cocoa drink (HFC; 609 mg CF/serving; *n* = 16) or low-flavanol cocoa drink (LFC; 13 mg CF/serving; *n* = 16) for 4 weeks. Assessment of HOMA-IR and ‘M’ during a 3-h, 60 mIU insulin·m^−2^·min^−1^ euglycemic clamp was performed before and after the intervention. Data are the mean (SD). Changes to HOMA-IR (HFC −0.003 (0.57); LFC −0.0402 (0.86)) and ‘M’ (HFC 0.99 (7.62); LFC –1.32 (4.88) µmol·kg^−1^·min^−1^) after the intervention were not different between groups. Four weeks’ consumption of ~1.2 g CF/day did not improve indices of fasting insulin sensitivity or insulin-mediated glucose uptake. A recommendation for dietary supplementation with cocoa flavanols to improve glycemic control is therefore not established.

## 1. Introduction

It is well established that overweight and obesity are risk factors for developing type 2 diabetes mellitus (T2DM). The progression from normal glucose regulation to T2DM usually involves the development of insulin resistance (IR), which in turn leads to impaired fasting glucose (IFG) and/or impaired glucose tolerance (IGT). There is interest in the impact that dietary interventions can have on preventing this transition, including awareness that some dietary phytochemicals may improve insulin sensitivity and thus reduce the risk of progression to T2DM [1,2].

In addition to this link between IR and T2DM, the pathologies of endothelial dysfunction and IR appear to co-exist, with both being risk factors in the development of cardiovascular disease [3,4]. Impaired endothelial function, characterized by a blunted vascular response to endothelium-dependent vasodilators, has been described in those who are obese, and it is suggested that the impaired cellular glucose uptake observed in these individuals is preceded and exacerbated by dysfunction in the microvasculature [5]. The intake of cocoa flavanols has been reported to acutely and chronically improve vascular function by increasing circulating nitric oxide [6,7], and in several prospective trials (in a range of cohorts), derived indices of fasting insulin sensitivity also seem to be improved by the consumption of cocoa-containing foods [8]. However, these observations are not unequivocal [8,9,10,11], and difficulties in blinding participants to interventions may be a confounding factor in some investigations [8,11]. Moreover, little focus has been given to the effect of cocoa consumption on ‘postprandial’ insulin sensitivity (sensitivity of tissues to insulin in the insulin-stimulated state) [12]. In the insulin-stimulated state, microvascular perfusion influences substrate delivery and glucose uptake into metabolizing tissues, with this tissue perfusion suggested to be negatively impacted in IR [13]. Thus, a role for cocoa flavanol dietary supplementation in enhancing tissue perfusion and increasing insulin-stimulated glucose uptake has been proposed [14]. Indeed, an improvement in the insulin sensitivity index (derived from an oral glucose tolerance test) [15] following 2 weeks’ daily consumption of dark chocolate in patient groups with hypertension has been described [16,17], although, in a similar cohort, Muniyappa et al. reported no improvements in insulin-mediated glucose uptake using a 2-h hyperinsulinemic clamp following 2 weeks of high-flavanol cocoa (HFC) beverage consumption [10].

Despite the paucity of primary data, multiple literature reviews have proposed a benefit of cocoa-containing foods in ameliorating ‘insulin resistance’ in humans, largely based on fasting, surrogate measures. Moreover, many protocols that have demonstrated a positive impact of cocoa-containing foods on measures of fasting insulin sensitivity have an initial run-in period where participants consume a diet that is low in flavonoids, which is maintained throughout any supplementation period, such that the intervention is on top of, and is compared to, a low-flavonoid background diet [16,17,18,19,20,21,22]. Results from testing under this dietary restriction may not reflect the impact of dietary supplementation in a real-world scenario, and, whilst useful to prove a principle or for mechanistic evaluations, it has limited utility for determining public health messages. Therefore, the purpose of the current study was to address some of the limitations in the existing literature by using the validated hyperinsulinemic clamp technique, and a double-blinded, placebo-controlled design, to determine the impact of HFC consumption on whole-body insulin sensitivity when supplemented on top of the habitual diet.

## 2. Materials and Methods

Trial design: A randomized, double-blinded, placebo-controlled, parallel-group study design was employed, with the random allocation sequence to receive either HFC or LFC generated using an online calculator [23]. The number of individuals in each group was balanced in 4 blocks (8 individuals per block; 4 HFC and 4 LFC) to promote participants of each group being on the trial at similar times of the year and mitigate any confounding effects of seasonal variation in habitual diet. Individuals were randomized at the point of entering the study, with their participant number allocated sequentially. Previous investigations investigating the cardiometabolic benefits of cocoa flavanol consumption have supplemented the diet for between 2 and 52 weeks. However, a meta-analysis indicated that the duration of intervention did not impact outcomes related to markers of fasting insulin resistance [8]. An intervention of 4 weeks was therefore chosen to allow participants to be assessed at the same point in their menstrual cycle.

This study was conducted according to the guidelines laid down in the Declaration of Helsinki 1973 (revised 1983) and all procedures involving human participants were approved by the University of Nottingham Medical School Ethics Committee. Written informed consent was obtained from all volunteers, and the protocol was registered at www.clinicaltrials.gov (reference NCT01201590). No changes were made to the protocol after trial commencement.

Participants: Pre-menopausal females who were overweight or grade 1 obese were recruited from the general Nottingham (UK) population through adverts placed in local newspapers. Those who fulfilled age (18–50 years), BMI (27–35 kg·m^−2^) and general health criteria at initial pre-screening attended a medical screening at the David Greenfield Human Physiology Unit (Medical School, Nottingham, UK) in the morning after an overnight fast to confirm health status and inclusion criteria. Those with a regular menstrual cycle (including those taking oral contraception medications) or using a hormonal intrauterine device without a monthly bleed were eligible for the study. The BMI inclusion range at pre-screening was designed to maximize the proportion having an elevated HOMA-IR but minimize the number exhibiting health problems that would exclude them from participating. HOMA-IR and QUICKI were determined according to standard methods [24,25] and individuals were accepted onto the trial if HOMA-IR was >1.5, they consumed caffeine containing foods/drinks on a daily basis, they did not report any food allergies related to the investigational product, they were not pregnant or breast feeding and they did not demonstrate any clinically significant abnormalities on screening.

Elevated HOMA-IR was a proxy to identify mild fasting IR and allow comparison with other studies, a value >1.5 having previously defined mild fasting insulin resistance in our laboratory. There was no upper limit for HOMA-IR, but a type 2 diabetes diagnosis based on fasting blood glucose concentration was set as an exclusion criterion. This cohort was identified as individuals at risk of developing T2DM, but who did not warrant pharmacological intervention. Eligibility criteria were not altered during the recruitment period.

Intervention: Study visits took place during the first 10 days of participants’ menstrual cycle, in the morning after an overnight fast, and approximately 12 h after the final cocoa drink had been consumed. For those using a hormonal intrauterine device (3 participants), study visits were scheduled a month apart. Participants were weighed and a dual-energy X-ray absorptiometry (DEXA) total body composition scan was made (Lunar Prodigy, GE Medical, Hatfield, UK) to address the potential confounder of body composition on insulin sensitivity measures. The percentage of total tissue that was fat mass was calculated for two regions (using manufacturer-defined parameters): the femoral–gluteal region (‘Gynoid’ distribution), which contains predominantly subcutaneous fat, and the abdominal region (‘Android’ distribution), which is composed of subcutaneous and visceral fat. Participants then rested semi-supine and an intravenous cannula (Becton Dickinson, Helsingborg, Sweden) was inserted retrograde into a dorsal hand vein for arterialized-venous blood sampling, and antegrade into a forearm vein for 0.5 IU·mL^−1^ insulin (Actrapid; Novo Nordisk, A/S.Bagsværd, Denmark) and 20% glucose (Baxter Healthcare Ltd., Thetford, UK) infusion. The cannulated hand was placed into a hot-air warming unit (air temperature 50–55 °C) for the duration of the study. A baseline blood sample was taken from the dorsal hand vein (via a 3-way tap) 20 min later to determine serum insulin and whole blood glucose concentration, and a 15 min ventilated hood indirect calorimetry (GEMNutrition Ltd., Daresbury, UK) measurement subsequently made to allow net substrate disappearance to be calculated according to standard methods [26].

A 3-h hyperinsulinemic (60 mIU·m^−2^·min^−1^), euglycemic (4.5 mmol·L^−1^) clamp was then commenced (t = 0 min) [27]. Arterialized venous samples were taken every 5 min for whole blood glucose and every 15 min for subsequent insulin analysis. Indirect calorimetry was further performed from t = 150 to 165 min. Glucose disposal was calculated from the amount of glucose infused and any change in blood glucose concentration over each 5 min period [27]. These values were standardized for body weight and averaged to give 15 min means, with steady-state glucose disposal rate (‘M’) calculated between t = 135 and t = 165 min.

Participants returned for the post-intervention study visit approximately a month later (depending on the length of their menstrual cycle), having consumed the investigational product twice daily over this time, and the protocol described above was repeated. Over the intervention period, participants were instructed not to change their normal lifestyle and to maintain their usual physical activity levels.

Investigational Product: The investigational products were dairy-based beverages made with either high-flavanol cocoa (609 mg cocoa flavanols, 95 mg (-)-epicatechin per serving; HFC group) or low-flavanol cocoa (13 mg of flavanols, 2 mg (-)-epicatechin per serving; LFC group). Products were supplied by Mars Incorporated (McLean, VA, USA) as powdered mixes, which were reconstituted by participants with 150 mL of warm water and consumed immediately following preparation. Powder sachets were identified by an anonymized code to blind volunteers and researchers to product allocation. The total amount of flavanols in each product was determined using the method described by Adamson et al. [28] and represents the sum of the monomeric flavanols and the oligomeric flavanols with a degree of polymerization up to and including 10. Flavanol content of each cocoa beverage was achieved by different processing methods and the drinks were similar with regard to macronutrient composition, flavor and color (Supplemental Table S1).

Participants were given a 5-week supply of beverages and the supplementation period began on the day after the first laboratory visit. They maintained a daily record of product consumption over the intervention (to assess intake compliance), and weekly telephone contact was maintained throughout to identify any problems.

Dietary Assessment: After recruitment, but before the first study visit, participants recorded all food intake, including snacks and drinks, in a 3-day (2 week and 1 weekend day) dietary record, using household measures to estimate portion size. Records were analyzed for daily macronutrient and energy intake using a food composition database (WISP V2, Tinuviel Software UK 2003), and an average over all 3 days of the recording period was taken. To reduce the impact of diet composition on the clamp-derived insulin sensitivity measures, the antecedent diet was then modified, by minor adjustments to the participant’s usual food choices, such that, in the 3 days prior to each study visit, 50% of the energy intake was provided by carbohydrates.

In the third week of supplementation, participants completed a second 3-day dietary record to allow any change in diet, as a result of the intervention, to be characterized. Individuals were made aware of the extra dietary energy provided by the investigational beverages and advised to replace their current snack intake with the drink but were instructed to make no other changes to their habitual diet.

Statistical Methods: Data were checked for normality of distribution and analyzed using SPSS version 25.0 (IBM, Armonk, NY, USA). Normally distributed data are described in the text as the mean with standard deviation in parentheses. Data that did not show a normal distribution are reported as the median with the 25th and 75th percentile in square brackets. Group characteristics pre-intervention were compared using unpaired Student’s t-test or non-parametric equivalent (Mann–Whitney U), and changes in variables at two time points but within groups were investigated using paired Student’s t-test or Wilcoxon signed ranks as appropriate. Cohen’s d was used to calculate standardized effect sizes [29]. Comparisons made between the 2 groups in variables measured at more than one time point employed a mixed-model ANOVA with repeated measures, with the partial eta squared value (ƞp^2^) calculated to estimate a standardized effect size. Statistical significance was assumed where p < 0.05.

Blinding of all parties to the randomization group was maintained until after the study had been completed and data had been statistically analyzed and interpreted by the research team.

Analytical Methods: Whole blood glucose was measured using the glucose oxidase enzymic method (Yellowsprings Inc., Yellow Springs, OH, USA; inter-assay and intra-assay coefficient of variation (CV) being 1%). Serum insulin was assessed using a human-specific radioimmunoassay (Merck Millipore, Billerica, MA, USA), with an intra-assay coefficient of variation (CV) of 4.41%, and an inter-assay CV of 10.53%.

Sample size: Statistical power was calculated using a primary end point of steady-state glucose disposal ‘M’, with expected M values in insulin-resistant individuals being between 16 and 30 µmol·kg^−1^·min^−1^ [30] and mean expected improvement in the HFC group being 33% [17]. Applying the variability of repeat assessment of ‘M’ reported by Bokeman et al. [31], 16 participants in each group (HFC vs. LFC control drink) were predicted to provide a statistical power of 80% [32].

## 3. Results

### 3.1. Participants

Interest was expressed by over 700 females, of whom 105 fulfilled the inclusion (except HOMA-IR) criteria and undertook a medical screening. Forty-four demonstrated HOMA-IR > 1.5 and were randomized into either the HFC or low-flavanol cocoa (LFC) group, with four withdrawing due to adverse effects, which were possibly related to the drinks, though not clearly related to the intake of cocoa flavanols per se, as adverse effects were reported in both groups (1× exacerbation of irritable bowel syndrome and diarrhea (LFC beverage), 3× headaches (reported in both LFC and HFC)). Eight others withdrew due to changes in circumstances (Supplementary Figure S1 Consort Diagram). Thirty-two participants completed the protocol and only data from these are reported. At screening, age, BMI, HOMA-IR and QUICKI were similar between groups (Table 1).

### 3.2. Compliance

The supplementation period varied between 26 and 36 days, depending on the length of the participant’s menstrual cycle, and self-reported compliance ranged from 81 to 100% (HFC 93.7 (5.3) %, LFC 93.3 (6.3) %; *p* = 0.866). Neither the supplementation period nor self-reported compliance influenced the findings when included as covariates in the statistical analysis (data not shown).

### 3.3. Insulin Sensitivity

**Figure 1:**
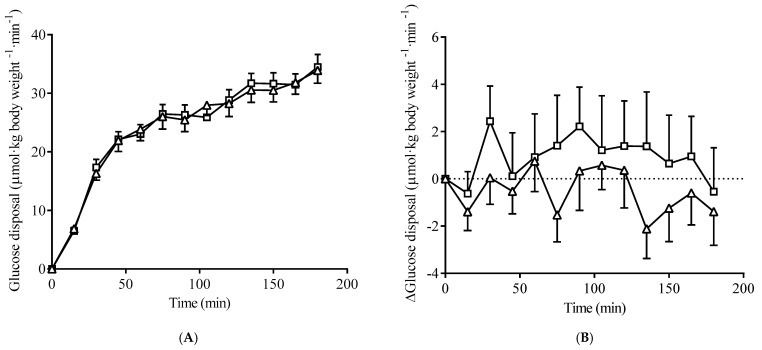
(**A**) Glucose disposal rate during the pre-intervention hyperinsulinemic clamp and (**B**) change in glucose disposal rate after the intervention (measured during the clamp), for the high-flavanol cocoa (□) and low-flavanol cocoa (Δ) groups (*n* = 16 in each group). Data are the mean, with error bars indicating SEM.

There were no differences in indices of fasting insulin resistance (HOMA-IR and QUICKI) between groups pre-intervention (Table 1). Steady-state glucose disposal during the clamp was achieved between 135 and 165 min, although uptake appeared to increase at the 180 min time point, as participants anticipated the end of the study (Figure 1A). Glucose disposal over the 3-h clamp, including the rate of increase in disposal observed in the first 60 min, was similar between groups pre-intervention. In the HFC group, the difference in glucose disposal post-intervention (compared to pre-intervention) was numerically above LFC (Figure 1B). However, the effect size of this observation was characterized as ‘trivial’ [33] (ƞp^2^ = 0.017) and was not statistically significant. Moreover, the change in M value across the intervention (ΔM: HFC 0.99 (7.62) µmol·kg^−1^·min^−1^; LFC −1.32 (4.88) µmol·kg^−1^·min^−1^; Cohen’s d = 0.36) and change in indices of fasting insulin resistance as a result of supplementation (ΔHOMA-IR; HFC −0.0031(0.566), LFC −0.0402 (0.861); Cohen’s d = 0.059 and ΔQUICKI; HFC 0.001 (0.047), LFC 0.009 (0.048); Cohen’s d = 0.047) were not different between groups.

### 3.4. Body Composition

Body weight was not different between the groups pre-intervention and did not change over the intervention (Table 2). Two individuals did not undergo a DEXA scan post-intervention and only those participants with a complete data set for fat-free mass (FFM) and percentage gynoid (%G) and android (%A) fat (n = 15 per group) are displayed. No differences in FFM or fat distribution were seen between groups pre-intervention. However, there was a significant visit*group interaction (*p* < 0.01) in the percentage of fat in the android region, with %A being lower in LFC after the intervention period (*p* < 0.05). Further analysis of the M value, clamp glucose disposal profile and HOMA-IR, using %A as a covariate, did not indicate that this variable was influencing the results. Moreover, changes in M (compared to pre-intervention), did not appear to be associated with changes in %A in the HFC (R^2^ = 0.0103) or the LFC group (R^2^ = 0.0293).

### 3.5. Indirect Calorimetry

**Figure 2:**
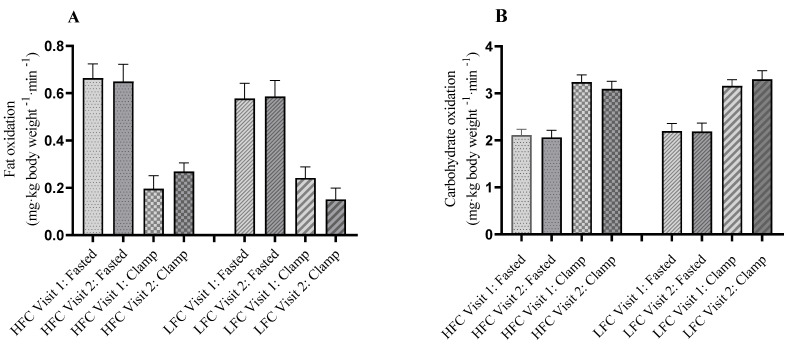
(**A**) Fat oxidation and (**B**) carbohydrate oxidation rates measured using indirect calorimetry before (Fasted) and at the end (150–165 min) of the hyperinsulinaemic clamp (‘Clamp’), PRE- and POST-intervention. Data for the high-flavanol cocoa (HFC; *n* = 14) and low-flavanol cocoa (LFC; *n* = 15) groups are the mean with SEM displayed as error bars.

Acquisition of indirect calorimetry data before the insulin infusion commenced and, during the steady-state period of the clamp, was not achieved in three participants due to technical difficulties. Therefore, data are reported from 14 participants in the HFC group and 15 in the LFC group. Mean fasting REE was approximately 59 kJ.kg^−1^·min^−1^ in both groups, on each visit, and did not change during the clamp (Table 2), suggesting that the insulin-stimulated uptake of glucose did not induce thermogenesis in these participants. Before the intervention period (‘PRE’), mean fat oxidation in the LFC group, in the fasted state, was approximately 10% lower than in the HFC group. However, this was not statistically significant or notable in terms of trends (Figure 2A). At the 150–165 min measurement (‘Clamp’), fat oxidation, in both groups, was suppressed by an average of 0.40 (0.26) kg^−1^·min^−1^ at PRE as a result of the insulin infusion. The intervention period did not appear to affect the insulin-stimulated decrease in fat oxidation in either group, with fat oxidation rates being similar between the groups when fasted and in the insulin-stimulated state across both PRE and POST intervention visits. Conversely, the insulin infusion stimulated carbohydrate oxidation, with a median increase of 50% above baseline values observed in both groups at the end of the clamp (Figure 2B). Carbohydrate oxidation rates were similar between the groups at baseline and in the clamp across both PRE and POST visits, and the intervention period did not affect the insulin-stimulated increase in carbohydrate oxidation.

### 3.6. Dietary Intake

Total dietary energy consumption, macronutrient intake and diet composition were similar between the groups at baseline (Table 3). For an overweight cohort, the reported energy intake was lower than would be expected, suggesting that some under-reporting might have occurred. However, the composition of the diet, with respect to the percentage of total energy intake provided by macronutrients, was representative of the average UK diet [34]. No variables changed significantly over the intervention period between groups, or within groups between visits. For the most part, participants appeared to modify their habitual diet, during the supplementation period, to compensate for the extra ~770 kJ consumed through drinking 2× cups of cocoa a day. This is reflected in the similar total energy intakes recorded in the diet diaries before and during the intervention period, and the absence of a change in body weight over the course of the study (Table 2).

## 4. Discussion

Overweight and obesity are associated with the condition of insulin resistance (IR) and impaired vascular function [5]. The reduced insulin-stimulated glucose uptake observed in these individuals may involve an impairment in insulin-mediated vasodilation and, by extension, reduced substrate delivery [35]. Consumption of HFC has been shown to improve vascular function acutely [36] and chronically [37,38]. However, despite a previously documented benefit of consuming the HFC cocoa product on vascular function [10], and the proposed relationship between endothelial dysfunction and impaired glucose uptake [14], twice-daily consumption of a HFC beverage (providing ~1.2 g of cocoa flavanols; 190 mg (-)-epicatechin a day) for 4 weeks in the current study did not improve glucose uptake in the insulin-stimulated state in females with overweight/obesity and mild insulin resistance, as characterized by the following: no increase in the rate of rise in whole-body glucose disposal during the first 30 min of the clamp; the absence of an improvement in M value; no enhancement of insulin-stimulated carbohydrate utilization. These outcomes are supported by other groups, including Balzer et al., who demonstrated that while cocoa consumption improved nitric oxide-dependent vascular function, fasting plasma glucose concentration, HbA1c and glycemic control remained unaffected in patients with T2DM [37]. Moreover, in older males, enhanced insulin-stimulated glucose uptake was not evident after acute HFC ingestion, despite a concurrent increase in muscle microvascular blood volume [39]. Therefore, it suggests that microvascular blood flow did not limit insulin-stimulated glucose uptake in the current cohort, and other molecular and metabolic impairments implicated in IR [40] are likely to be predominating.

In opposition to the current results, a calculated index of postprandial insulin sensitivity (ISI) derived from the oral glucose tolerance test [15] increased in individuals following dietary supplementation with dark chocolate [16,17]. Differences in the supplementation product (cocoa beverage vs. chocolate [41]) and study design (±dietary flavonoid restrictions, blinding vs. non-blinding of investigational product) may contribute to the contradictory outcomes. However, inferences made about whole-body insulin sensitivity based on oral feeding methods can be influenced by changes in gastric emptying rate and glucose absorption across the gut [42], such that improvements in ISI may not merely reflect a change in tissue insulin sensitivity. In contrast, the hyperinsulinemic clamp provides a standardized, reproducible method for assessing whole-body glucose uptake in the insulin-stimulated state, without the potential confounding factors associated with the ISI.

The mechanisms underpinning IGT and IFG are different [43,44,45]. Therefore, an intervention that does not impact insulin-stimulated glucose uptake might still have a beneficial effect on indices of fasting insulin sensitivity. However, in the current study, the intervention had a ‘trivial’ (as defined by Cohen [29] and others [33]) effect on HOMA-IR, suggesting that the absence of a difference was not simply due to insufficient statistical power. Other groups have also failed to detect an improvement in HOMA-IR following a period of cocoa or chocolate consumption [10,11,46,47], whilst reductions in HOMA-IR have been described following dietary supplementation with dark chocolate [16,17,18] and HFC beverages [21,22], driven both by reductions in blood glucose alone [21] and together with reduced circulating insulin [17,18,22]. Whilst it is acknowledged that variation between insulin assays means that direct comparison of HOMA-IR values between studies is not always appropriate, differences in the degree of fasting insulin resistance demonstrated by different study cohorts theoretically could impact the findings. The participants in the current study had a median HOMA-IR of 2.25. However, other studies have included individuals whose HOMA-IR values were higher [16] and, potentially, the ability to elicit an improvement in fasting insulin sensitivity could be enhanced where the impairment is greater [8]. Indeed, in a study investigating the vasoactive effects of dark chocolate consumption on those with hypertension, the largest improvement was seen in those with HOMA-IR values greater than 5 [17]. However, a reduction in HOMA-IR has been reported after 15 days of dark chocolate consumption in individuals without hypertension, and these participants exhibited HOMA-IR values that were similar to, or lower than, those recorded in the current study [18]. It is therefore unclear whether the initial HOMA-IR value in the current study participants per se was a limitation.

The absence of an improvement in insulin resistance measures in this study may have been influenced by the mitigating effects of estrogen on measurements in the pre-menopausal female participants [48]. However, the study visits were scheduled to test participants at a similar point in their menstrual cycle to control for hormone fluctuations, and whilst it is possible that a male cohort might have responded differently to the intervention, a systematic review collating the impact of cocoa consumption on insulin resistance measures did not detect a difference in outcomes between male and female participants [8].

An important strength of the current study was that the control (LFC) drink was matched to the HFC drink with regard to taste and color, such that participants and study personnel were effectively masked to treatment allocation. In contrast, other studies report using white chocolate as the control for a dark chocolate test product or using milk as the control for a cocoa beverage [16,18,19,20]. In these instances, the macronutrient profile, taste and appearance were very different, and this lack of effective blinding could have influenced findings [18,43,49].

Finally, most protocols that have previously demonstrated a positive impact of cocoa-containing foods on measures of insulin sensitivity assess the impact of supplementation on top of, and compared to, a low-flavonoid background diet [16,17,18,19,20,21,22]. In contrast, the present study did not modify the baseline diet composition and investigated the impact of adding >1 g of cocoa flavanols per day on top of the habitual diet. Importantly, the supplementation protocol was chosen to reflect usual dietary supplementation behaviors in the UK, as the ability to show the efficacy of a nutritional component under these conditions would be central when determining public health messages. It is possible that the absence of an effect of cocoa consumption on measures of insulin sensitivity in the current study was due to an insufficient stimulus when cocoa flavanols were provided alongside a diet that potentially already contained bioactive compounds. However, the supplementation regime provided approximately six times the amount of epicatechins found in the average UK diet and approximately tripled the daily total flavonoid intake [50]. This increase in intake is of a similar or greater magnitude compared to other studies that have supplemented with smaller amounts of cocoa flavanols against a low-flavonoid background diet [8]. Therefore, it is believed that the supplementation regime of the current study was a sufficient stimulus to test any benefit of dietary supplementation with cocoa flavanols on insulin sensitivity.

## 5. Conclusions

In summary, supplementing the diet of females with overweight/obesity with an HFC beverage for 4 weeks did not improve HOMA-IR or insulin-stimulated glucose disposal. These findings arise from both standard fasting measurements and the rigorous hyperinsulinemic clamp method and support the conclusion that evidence to recommend cocoa flavanol dietary supplementation to improve glycemic control is currently insufficient [11,12]. This highlights the need for data to be available from well-controlled prospective studies, which mimic usual dietary supplementation behaviors, to allow coherent public health messages to be devised.

## Figures and Tables

**Table 1:** Parameters at initial screening in those subsequently randomized to high-flavanol (HFC) or low-flavanol (LFC) cocoa drink. Data show the mean with SD in parentheses for normally distributed data, or the median with 25th–75th percentile in square brackets where distribution did not meet normality criteria.

	HFC (*n* = 16)	LFC (*n* = 16)
Mean Age (years)	31.9 (11.20)	34.8 (9.13)
Mean BMI (kg/m^2^)	30.6 (2.52)	31.7 (2.17)
Median HOMA-IR	2.25 [1.77–3.81]	2.25 [1.78–3.67]
Mean QUICKI	0.334 (0.017)	0.336 (0.016)

**Table 2:** Measures made pre- (PRE) and post-intervention (POST) in those consuming high-flavanol (HFC) or low-flavanol (LFC) cocoa.

	HFC		LFC	
	PRE	POST	PRE	POST
Body Weight (kg)	85.45 (8.18)	85.74 (8.86)	85.73 (8.07)	86.59 (8.33)
FFM (kg)	37.17 (3.78)	37.05 (4.46)	37.24 (3.21)	37.80 (3.06)
^#^ Gynoid Fat (%)	52.7	53.4	54.4	52.5
	[50.0–54.9]	[49.4–55.5]	[49.7–55.4]	[48.0–55.5]
^#^ Android Fat (%)	56.0	55.6	55.2	54.4 *
	[52.6–58.5]	[53.4–58.8]	[50.5–58.1]	[49.6–58.4]
^#^ Fasting Insulin (pmol·L^−1^)	39.9	49.8	37.8	31.8
	[26.4–57.6]	[34.7–67.4]	[28.5–67.4]	[20.1–79.2]
M Value (µmol·kg^−1^·min^−1^)	31.0 (7.87)	31.6 (6.93)	32.0 (9.54)	30.3 (7.83)
REE: Fasted (kJ/day)	7104 (776)	7033 (1015)	6979 (691)	7050 (625)
REE: End of the Clamp (kJ/day)	7104 (612)	7050 (896)	7183 (652)	7100 (873)

*n* = 16 in each group at each visit except for dual-energy X-ray absorptiometry measures (fat-free mass (FFM), and percentage of total tissue that is fat in the gynoid and android regions), where data are for *n* = 15 in each group at each visit. Resting energy expenditure (REE) was measured before (fasted) and at the end of the clamp (150–165 min). M value indicates steady-state glucose uptake. Data show the group means with SD in parentheses, and medians ^#^ are displayed with the 25th and 75th percentiles in square brackets. * *p* < 0.05 compared to PRE.

**Table 3:** Macronutrient dietary intake pre- and in week 3 of the supplementation period. Median [25th–75th percentile] daily values were derived from diet diaries measured on 2× week (working) and 1× weekend (rest) day.

	HFC		LFC	
	PRE	Week 3	PRE	Week 3
Protein (g)	76.1	70.4	68.9	73.3
	[65.5–82.2]	[58.8–78.2]	[61.4–77.0]	[66.9–83.4]
Fat (g)	76.1	69.8	75.5	74.4
	[61.3–90.6]	[58.4–78.0]	[54.3–85.5]	[66.5–87.4]
CHO (g)	221.6	209.2	229.0	224.6
	[180.6–257.3]	[205.7–230.3]	[178.5–289.1]	[200.4–259.4]
Total sugars (g)	89.8	77.9	95.8	83.6
	[60.1–101.8]	[69.1–97.2]	[61.2–117.1]	[64.8–97.6]
Alcohol (g)	7.5 [0–12.0]	1.0 [0–12.0]	0 [0–7.0]	7.0 [0.5–16.0]
Total energy (E) (kJ)	7401	7368	8050	7803
	[7037–8803]	[6711–8694]	[6648–8903]	[7393–8828]
% of E derived from protein	16.1	16.1	15.3	16.1
	[15.0–18.2]	[14.2–17.6]	[13.0–17.5]	[13.8–17.3]
% E from fat	39.9	36.4	37.1	35.3
	[32.6–41.4]	[32.6–38.5]	[32.9–42.6]	[32.0–40.6]
% E from CHO	42.3	44.3	44.2	43.2
	[38.0–46.8]	[40.7–49.1]	[39.5–52.0]	[40.0–49.0]
% E from sugars	17.0	17.8	18.9	16.5
	[14.1–20.9]	[15.9–20.2]	[15.8–21.4]	[12.6–20.1]
% E from alcohol	2.9 [0–4.6]	0.5 [0–5.1]	0 [0–2.2]	2.4 [0.3–7.2]

## Data Availability

Data is deposited in the University of Nottingham open access data repository: https://rdmc.nottingham.ac.uk/ (DOI: 10.17639/nott.7268).

## Supplementary Materials

The following supporting information can be downloaded at: https://www.mdpi.com/article/10.3390/nu15030565/s1, Table S1: Nutritional content of the investigational products; Figure S1: Consort diagram.
