# Supplementary material for: Cocoa Flavanol Supplementation and the Effect on Insulin Resistance in Females Who Are Overweight or Obese: A Randomized, Placebo-Controlled Trial
Source: Nutrients. 2023 Jan 21;15(3):565. doi: 10.3390/nu15030565 (PMC9921219; doi:10.3390/nu15030565)
Supplement: Supplementary file 1 [file nutrients-15-00565-s001.zip › nutrients-2072667-supplementary.pdf]

Supplemental Figure S1: Consort Flow Diagram

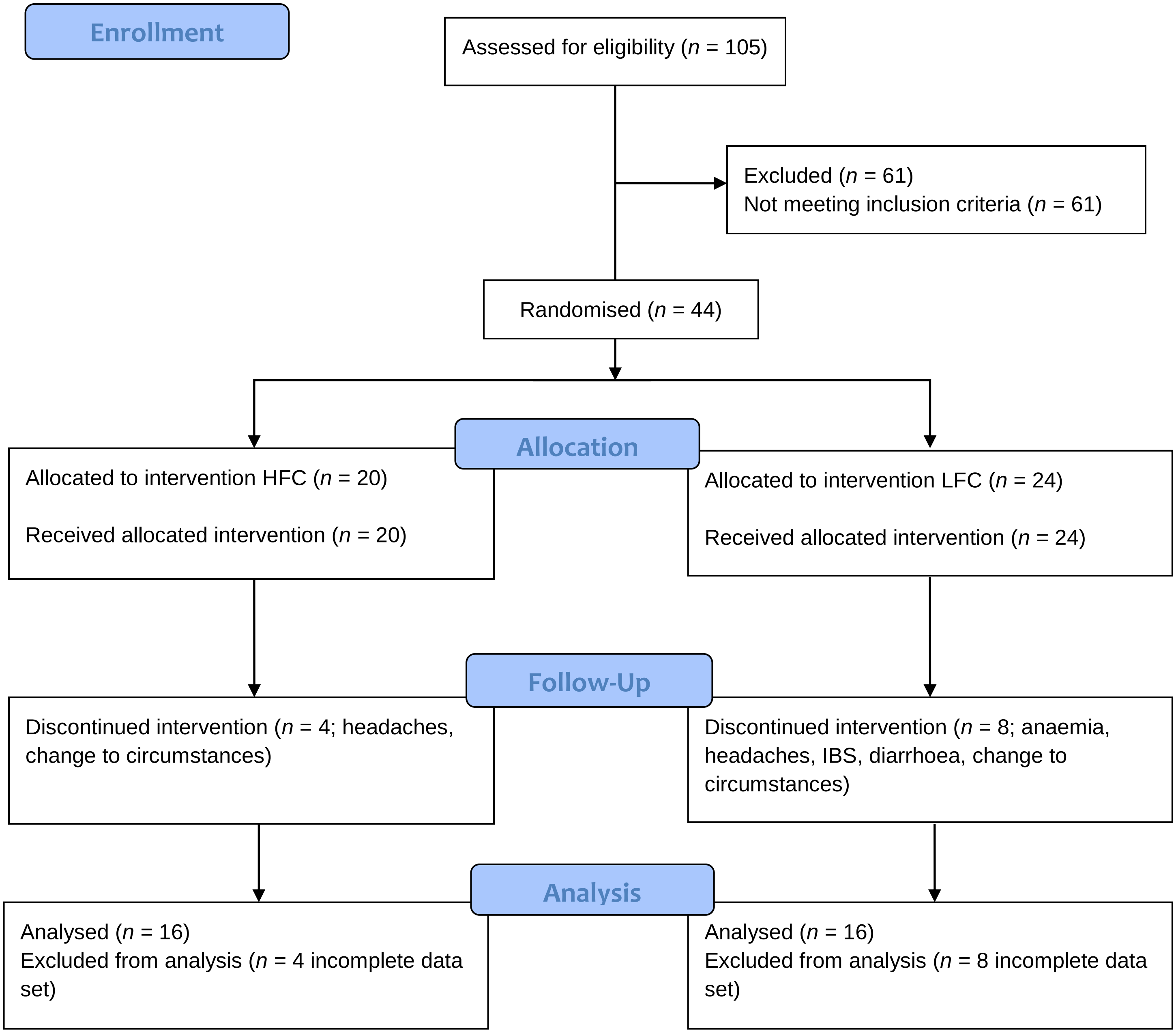

Supplementary Table S1: Nutritional content of the investigational products (information provided by manufacturer).*Determined according to Adamson *et al* (JAFC 1999; 47(10): 4184-4188).**Determined according to Machonis *et al* (J. AOAC Int. 2014;97(2): 506-509).

	**HFC**	**LFC**
Energy (kJ)	385	381
Total fat (g)	1.0	1.1
Saturated fat (g)	0.5	0.6
Cholesterol (mg)	4.0	4.0
Total Carbohydrates (g)	11.7	13.3
Sugars (g)	7.7	7.4
Protein (g)	7.2	7.0
Dietary Fibre (g)	2.1	2.6
Copper (mg)	0.3	0.3
Magnesium (mg)	64	59
Zinc (mg)	1.4	1.3
Sodium (mg)	87	114
Potassium (mg)	473	546
Calcium (mg)	182	185
Phosphorus (mg)	211	203
Caffeine (mg)	15	14
Theobromine (mg)	204	185
Total Cocoa Flavanols DP 1-10* (mg)	609	13
DP 1 **<br>(−)-epicatechin (mg)<br>(±)-catechin (mg)	 95<br>14	 2.0<br>1.7
